# The value of routine endoscopic ultrasound in patients with esophageal cancer undergoing active surveillance after neoadjuvant chemoradiotherapy

**DOI:** 10.1055/a-2776-5896

**Published:** 2026-02-12

**Authors:** Sanjiv S. G. Gangaram Panday, Matteo Pittacolo, Sjoerd M. Lagarde, Bianca Mostert, Judith Honing, J. Jan B. van Lanschot, Tanya M. Bisseling, Erik J. Schoon, Jolanda M. van Dieren, Rutger Quispel, Liekele E. Oostenbrug, Andries van der Linden, Sietske Corporaal, Lieke Hol, Eva Kouw, Jurjen J. Boonstra, Wouter L. Hazen, Erik Vegt, Manon C. W. Spaander, Bas P. L. Wijnhoven, on behalf of the SANO Study Group*

**Affiliations:** 1 Department of Surgery 6993 Erasmus MC Cancer Institute, University Medical Center RotterdamRotterdamThe Netherlands; 2 Department of Surgery, Oncology and Gastroenterology 9308 University of PaduaPadovaItaly; 3 Department of Medical Oncology 6993 Erasmus MC Cancer Institute, University Medical Center RotterdamRotterdamThe Netherlands; 4 Department of Gastroenterology and Hepatology 6993 Erasmus MC, University Medical Center RotterdamRotterdamThe Netherlands; 5 Department of Gastroenterology and Hepatology 6034 Radboud University Medical CenterNijmegenThe Netherlands; 6 Department of Gastroenterology and Hepatology 3168 Catharina HospitalEindhovenThe Netherlands; 7 Department of Gastrointestinal Oncology 1228 The Netherlands Cancer InstituteAmsterdamThe Netherlands; 8 Department of Gastroenterology and Hepatology 84744 Reinier de Graaf GasthuisDelftThe Netherlands; 9 Department of Gastroenterology and Hepatology 3802 Zuyderland Medical CentreHeerlenThe Netherlands; 10 Department of Gastroenterology and Hepatology 1153 ZGT HospitalAlmeloThe Netherlands; 11 Department of Gastroenterology and Hepatology 4480 Frisius MC LeeuwardenLeeuwardenThe Netherlands; 12 Department of Gastroenterology and Hepatology 7000 Maasstad HospitalRotterdamThe Netherlands; 13 Department of Gastroenterology and Hepatology 72485 Gelre HospitalsApeldoornThe Netherlands; 14 Department of Gastroenterology and Hepatology 4501 Leiden University Medical CenterLeidenThe Netherlands; 15 Department of Gastroenterology and Hepatology 7898 Elisabeth Tweesteden HospitalTilburgThe Netherlands; 16 Department of Radiology and Nuclear Medicine 6993 Erasmus MC, University Medical Center RotterdamRotterdamThe Netherlands

## Abstract

**Background:**

Active surveillance for esophageal cancer after neoadjuvant chemoradiotherapy (nCRT) involves repeated diagnostic tests to detect cancer regrowth. In the SANO trial, this included esophagogastroduodenoscopy (EGD) with biopsies, endoscopic ultrasound (EUS) with fine-needle aspiration (FNA) of suspicious lymph nodes, and fluorodeoxyglucose positron emission tomography with computed tomography (PET-CT). The value of routine EUS in this setting remains largely unknown. This study aimed to assess the diagnostic yield of EUS over PET-CT.

**Methods:**

A retrospective analysis of patients with esophageal cancer who underwent nCRT followed by clinical response evaluations with EGD, EUS, and PET-CT was performed. Initial response assessment was performed within 3 months post-nCRT. Patients without tumor regrowth underwent active surveillance with repeated diagnostic testing. The primary outcome was the rate of EUS-detected lymph node metastases missed by PET-CT, after excluding cases with positive EGD findings or distant metastases.

**Results:**

327 patients underwent both PET-CT and EUS post-nCRT, accounting for 1006 combined procedures: 327 at initial response assessment; 679 during active surveillance (6–60 months post-nCRT) in 121 patients. Positive lymph nodes were detected by EUS in 3.7% (12/327) of initial response assessments, with 2.1% (7/327) unidentified by PET-CT. During surveillance, this dropped to 0.9% of assessments (6/679), with 0.1% (1/679) missed by PET-CT.

**Conclusions:**

EUS with FNA adds most value at 3 months post-nCRT, when the likelihood of detecting recurrence is highest. Beyond 3 months, its added value is limited (0.1% with negative PET-CT). Restricting the use of EUS to PET-suspicious nodes could omit 98% of EUS procedures.

## Abstract (de)

**Graphical Abstract:**

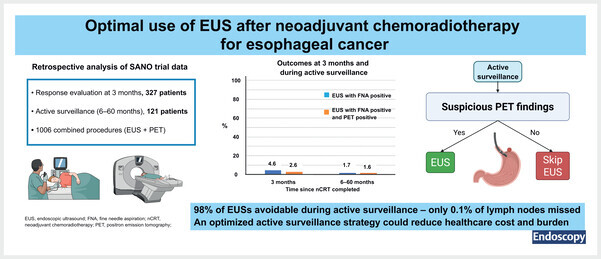

## Introduction

Multimodal management of esophageal and gastroesophageal junctional cancer traditionally involves perioperative chemotherapy or neoadjuvant chemoradiotherapy (nCRT) plus surgery. The option of active surveillance in patients who have complete clinical response (CCR) 12 weeks after nCRT seems valid, based on the recent SANO trial, which showed that 2-year overall survival in patients who underwent active surveillance was noninferior to standard surgery 1 .

The timely detection of cancer regrowth is important to ensure the selection of patients who still need surgery for locoregional residual disease after nCRT. According to the SANO protocol, local regrowth is assessed by esophagogastroduodenoscopy (EGD) with multiple bite-on-bite biopsies of the original tumor bed 2 . The combination of radial and linear endoscopic ultrasound (EUS) with fine-needle aspiration (FNA) can detect cancer regrowth in suspicious lymph nodes in up to 10% of patients who had negative biopsies 3 4 5 . Fluorodeoxyglucose positron emission tomography with computed tomography (FDG PET-CT) is mainly used for the detection of interval metastases, but may also be used to guide the endoscopist in selective FNA of suspicious lymph nodes.

In the first few months after nCRT, PET-CT cannot distinguish inflammatory changes in the esophagus or regional lymph nodes from residual or recurrent cancer, which may result in false-positive test results 6 7 . EUS has a higher sensitivity and specificity for detecting lymph node involvement, but its accuracy varies depending on lymph node location and operator experience. EUS has not been found to be useful for the detection of cancer regrowth based on measurement of wall thickness of the esophagus after nCRT; however, it might be useful when specific cutoff values are applied 3 8 .

Based on the preSANO study, combining PET-CT and EUS was considered optimal, as PET-CT can reveal suspicious lymph nodes missed by EUS, because EUS detected only 50% of malignant nodes 10–12 weeks after nCRT 9 . The combination of PET-CT and EUS is however costly and invasive, with EUS being operator-dependent and burdensome for the patient. Therefore, it is important to evaluate whether EUS could be reserved for selected indications, instead of being used routinely. To optimize active surveillance, it is important to assess whether PET-CT, despite its high false-positive rate, has a sufficiently low false-negative rate in the detection of regional tumor-positive lymph nodes. If so, EUS with FNA could be reserved for patients with FDG-avid lymph nodes on PET-CT, thereby reducing patient burden and healthcare costs.

This study aimed to evaluate the diagnostic yield of EUS in identifying nodal disease not detected by PET-CT following nCRT for esophageal cancer, and to assess the yield of EGD with biopsies, EUS with FNA, and PET-CT in detecting locoregional regrowth.

## Methods

### Study design and patients

A retrospective analysis was performed on patients with esophageal or gastroesophageal junctional cancer included in the multicenter SANO trial, who underwent nCRT followed by response evaluations and active surveillance with a minimum follow-up of 3 years. Inclusion criteria were: an FDG-avid tumor on the diagnostic (pretreatment) FDG PET-CT, and PET-CT combined with EUS during a response evaluation after nCRT 2 . As the study used data from the SANO trial, which had received ethical approval from the medical ethics committee of Erasmus MC (MEC-2017–392), no additional approval was required.

### Procedures

Patients underwent two clinical response evaluations (CREs): at 4–6 weeks (CRE-1) and 10–12 weeks (CRE-2) after nCRT. If no residual tumor was detected at CRE-1 and CRE-2, patients underwent active surveillance or standard surgery according to the SANO protocol.

CRE-1 involved an EGD with at least four bite-on-bite biopsies from the primary tumor site; CRE-2 included an EGD with bite-on-bite biopsies, EUS with FNA if there were suspicious lymph nodes, and PET-CT. PET-CT was scheduled before EUS, so that the PET-CT images could be used to guide the EUS and help to identify suspicious lymph nodes.

In patients undergoing active surveillance in the SANO trial, EGD, EUS, and PET-CT were repeated at 6, 9, 12, 16, 20, 24, 30, 36, 48, and 60 months post-nCRT. Surgery was offered to the patient if residual or recurrent tumor was histologically confirmed or highly suspected, in the absence of distant metastases. Patients with distant metastases, with or without locoregional cancer regrowth, discontinued active surveillance. Procedures were included up to and including the detection of distant metastases. During EUS, FNA was the standard approach. Fine-needle biopsy (FNB) was performed only incidentally, at the discretion of the endoscopist when additional tissue was needed. All EUS procedures were performed by experienced specialist gastroenterologists from dedicated esophageal cancer centers, each with substantial expertise in EUS for esophageal cancer.

### Outcomes

The primary outcome was the percentage of procedures with residual tumor detected by EUS with FNA that had not been suspicious on PET-CT. This was analyzed for CRE-2 and also for later time points during active surveillance.

Secondary outcomes included the yield of other diagnostic modalities, EGD with bite-on-bite biopsies, EUS with FNA, and PET-CT, for the detection of locoregional regrowth, defined as local vital tumor at the primary tumor site or regional lymph node metastases, in absence of distant metastases. The following definitions were used: “local” referred to the primary tumor site, “regional” referred to the regional lymph nodes, and “locoregional” referred to the combination of the primary tumor and regional lymph nodes.

A suspicion of lymph node metastasis on PET-CT was defined as lymph nodes showing FDG uptake above background on PET-CT (i.e. more than minimal physiological uptake) or being described as suspicious in the radiology report. Suspicious lymph nodes on EUS were defined as round, hypoechoic, >5 mm, or showing FDG uptake on PET-CT. FNA was performed at the endoscopist’s discretion when a node was considered suspicious. A positive EUS with FNA was defined as the presence of tumor cells on cytological assessment of the aspirate. In cases where there was uncertainty owing to nonrepresentative material or inconclusive cytology, the outcome of the subsequent EUS was used. If uncertainty remained or if FNA was not/could not be performed, EUS was considered negative for this analysis, as it did not provide additional value in detecting tumor regrowth and did not lead to surgery. For lymph nodes with FDG uptake that were negative on EUS-FNA, the EUS-guided FNA results were considered definitive, as PET-CT may show false positives from inflammation. Patients with high suspicion for regrowth on PET-CT who underwent resection were discussed in the multidisciplinary tumor board prior to surgery.

### Statistical analysis

Frequencies and percentages were used to describe baseline clinical and tumor characteristics, patients with positive and negative PET-CT and EUS findings, and the diagnostic methods used to detect locoregional regrowth. Proportions of outcomes are presented with exact 95%CIs, with those below 10% reported to one decimal place. We excluded combined PET-CT and EUS procedures in which EGD with bite-on-bite biopsies had already detected local regrowth or PET-CT had already identified metastases (at the same CRE) to determine the true benefit of EUS during repeated CREs. In a subanalysis, we included those cases to assess how many EUS procedures could potentially be spared.

At CRE-2, we calculated the number needed to screen (NNS) based on a single assessment per patient; however, because patients may undergo multiple EUS procedures during active surveillance (beyond CRE-2), calculating a traditional NNS by combining all procedures would overestimate the screening burden and underestimate the true NNS. Therefore, we have reported diagnostic yield per patient and per procedure separately. To estimate the potential benefit of a selective EUS strategy based on PET-CT findings, we constructed a hypothetical cohort of 1000 patients at the CRE-2 timepoint and a second cohort undergoing active surveillance, accounting for PET positivity over time. Assumptions regarding detection rates by PET-CT and EUS were based on observed data.

Data were analyzed using R version 4.2.2 using the binom package and base R functions for descriptive statistics (R: A Language and Environment for Statistical Computing; The R Foundation for Statistical Computing, Vienna, Austria).

## Results

**Fig. 1:**
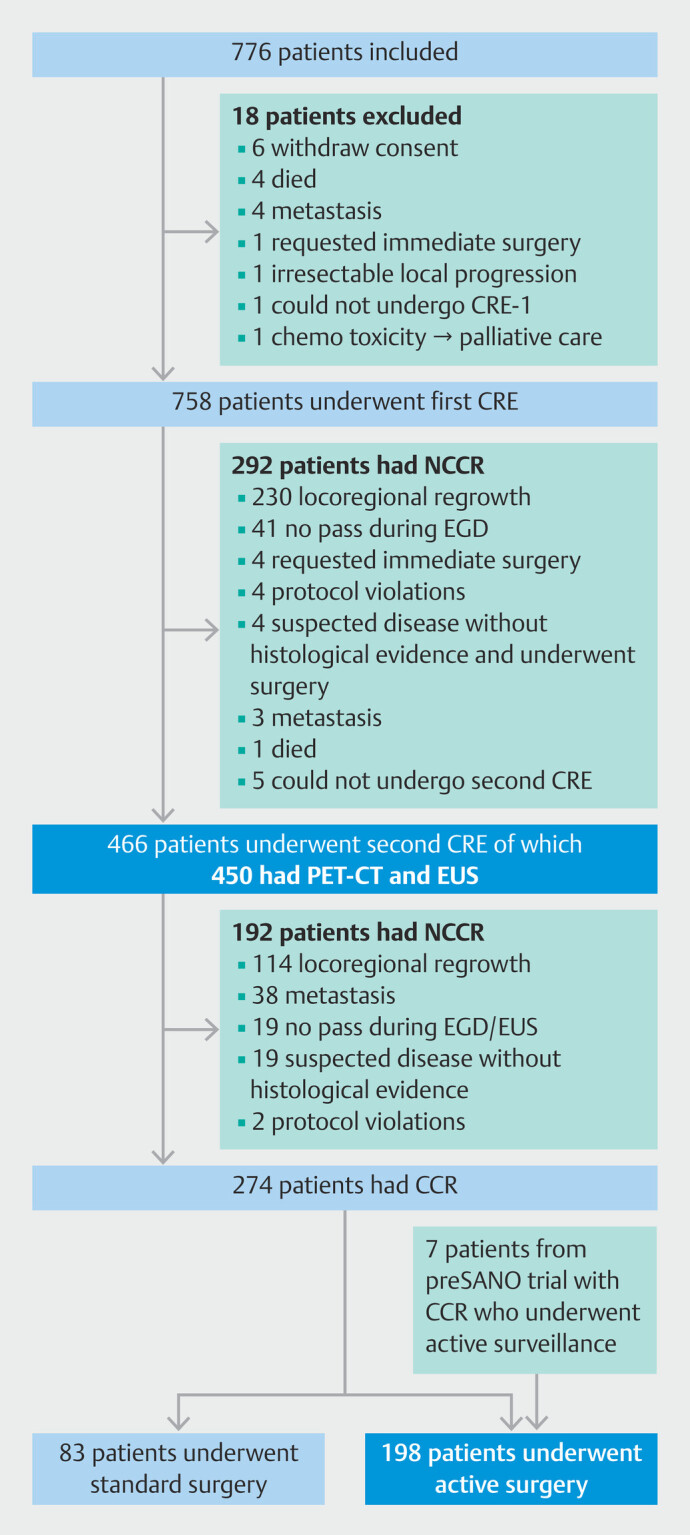
Patient flowchart. CCR, clinical complete response; CRE, clinical response evaluation; EGD, esophagogastroduodenoscopy; EUS, endoscopic ultrasound; NCCR, non-CCR; nCRT, neoadjuvant chemoradiotherapy; PET-CT, positron emission tomography with computed tomography.

A total of 466 patients underwent at least one diagnostic modality at CRE-2, of whom 450 patients had a combination of PET-CT and EUS ( Fig. 1 ). Of the 198 patients who underwent active surveillance, 179 patients had a total of 782 response assessments with PET-CT and EUS between 6 and 60 months after nCRT, with all patients having a minimum follow-up of 3 years. In total, 1232 response assessments were documented ( **Table 2s** , see online-only Supplementary material).

Baseline characteristics are presented in Table 1 . After the exclusion of combinations where EGD with bite-on-bite biopsies had already detected regrowth and/or PET-CT had identified metastases, 1006 procedures remained, including 327 at CRE-2 in 327 patients and 679 during active surveillance in 121 patients (beyond CRE-2).

**Table:** **Table 1** Baseline characteristics of all included patients.

	PET and EUS at CRE-2 (n = 450)	Active surveillance (n = 198)
Age, median (IQR), years	68 (62–73)	69 (63–74)
Sex, male, n (%)	363 (81)	156 (79)
Tumor histology, n (%)		
Adenocarcinoma	352 (78)	147 (74)
Squamous cell carcinoma	83 (18)	47 (24)
Other	15 (3)	4 (2)
Tumor differentiation grade, n (%)		
G1	72 (16)	33 (17)
G2	204 (45)	97 (49)
G3	163 (36)	65 (33)
Gx	11 (2)	3 (2)
Clinical T category, n (%) ^1^		
cT1	3 (1)	2 (1)
cT2	93 (21)	47 (24)
cT3	328 (73)	145 (73)
cT4	9 (2)	3 (2)
cTx	17 (4)	1 (1)
Clinical N category, n (%) ^1^		
cN0	174 (39)	86 (43)
cN1	166 (37)	77 (39)
cN2	83 (18)	31 (16)
cN3	8 (2)	4 (2)
cNx	19 (4)	0 (0)
WHO performance status, n (%) ^2^		
0	291 (65)	133 (67)
1	121 (27)	54 (27)
2	2 (0)	2 (1)
3	1 (0)	1 (1)
Missing data	35 (8)	8 (4)
Tumor location, n (%) ^3^		
Proximal esophagus	4 (1)	3 (2)
Mid esophagus	44 (10)	24 (12)
Distal esophagus	269 (60)	120 (61)
Esophagogastric junction	133 (30)	51 (26)
CRE, clinical response evaluation; EUS, endoscopic ultrasound; IQR, interquartile range; PET, positron emission tomography; WHO, World Health Organization. ^1^ cTNM staging is reported according to the 7th edition of the Union for International Cancer Control staging manual, with T staging and clinical nodal staging based on endoscopic ultrasonography or computed tomography. ^2^ WHO performance score, assessed on a 5-point scale, with higher numbers indicating greater disability. ^3^ Determined by endoscopy.		

EGD with bite-on-bite biopsies was performed in 758 patients at CRE-1 and 457 patients at CRE-2. Between 6 and 60 months after nCRT, 185 patients underwent 811 EGDs with bite-on-bite biopsies ( **Table 2s** ).

### Lymph node detection by EUS with FNA

EUS with FNA identified positive lymph nodes in 1.8% of all procedures (18/1006; 95%CI 1.1%–2.8%), including 0.8% of the procedures that missed positive lymph nodes by PET-CT (8/1006; 95%CI 0.3%–1.6%). At CRE-2, positive lymph nodes were detected by EUS with FNA in 3.7% of procedures (12/327; 95%CI 1.9%–6.3%), with positive lymph nodes missed in 2.1% of the procedures by PET-CT (7/327; 95%CI 0.9%–4.4%). During active surveillance, EUS with FNA identified positive lymph nodes in 0.9% of procedures (6/679; 95%CI 0.3%–1.9%) and in 0.1% of the procedures positive lymph nodes were missed by PET-CT (1/679; 95%CI 0.0%–0.8%) ( Table 2 ).

**Table:** **Table 2** Comparison of PET-CT lymph node uptake and EUS (with FNA) outcome for lymph nodes at CRE-2 and during active surveillance ^1^ .

	EUS + FNA positive	EUS negative ^2^	Total
All PET + EUS			
PET LN positive ^3^	10 (1.0%)	31 (3.1%)	41
PET LN negative	8 (0.8%)	957 (95.1%)	965
Total	18	988	1006
CRE-2			
PET LN positive ^3^	5 (1.5%)	21 (6.4%)	26
PET LN negative	7 (2.1%)	294 (89.9%)	301
Total	12	315	327
Active surveillance			
PET LN positive ^3^	5 (0.7%)	10 (1.5%)	15
PET LN negative	1 (0.1%)	663 (97.6%)	664
Total	6	673	679
CRE-2, clinical response evaluation at 10–12 weeks; CT, computed tomography; EUS, endoscopic ultrasound; FNA, fine-needle aspiration; LN, lymph node; PET, positron emission tomography. ^1^ Patients already positive for local regrowth on esophagogastroduodenoscopy and/or for distant metastasis on PET-CT were excluded. ^2^ Assumed negative as EUS did not aid in detection (PET suspicious LN: no/yes): –12 no FNA performed with suspicious lymph nodes (5 no, 7 yes) – 29 nonrepresentative material (28 no, 1 yes) – 2 uncertain cytology (2 no). ^3^ PET LN positivity indicates suspicion.			

### Yield of the different modalities for detection of locoregional regrowth

At CRE-1, local regrowth was detected in 230/758 patients (30%, 95%CI 27%–34%) by EGD with bite-on-bite biopsies. At CRE-2, locoregional regrowth was detected in 114/466 patients (13 patients with lymph node metastases) ( Table 3 ). In the active surveillance group, locoregional regrowth was detected in 100/198 patients (51%) after a median (IQR) follow-up of 54 months (46–58 months) ( Table 4 ).

**Table:** **Table 3** Detection of locoregional regrowth in patients at CRE-1 and CRE-2 ^1^ .

	Cases with locoregional regrowth detected, n	Diagnostic method
CRE-1	230	EGD with bite-on-bite biopsies
CRE-2	114	EGD with bite-on-bite biopsies (n = 101) PET positive (n = 86) ^2^ PET negative (n = 15) ^3^
		EUS/PET (n = 13) LNs detected by EUS + FNA, PET LN positive (n = 5) ^4^ LNs detected by EUS + FNA, PET LN negative (n = 7) LNs detected by EBUS + FNA, PET LN positive (n = 1)
CRE, clinical response evaluation; nCRT, neoadjuvant chemoradiotherapy; EGD; esophagogastroduodenoscopy; E(B)US, endoscopic (bronchial) ultrasound; FNA, fine-needle aspiration; LN, lymph node; PET, positron emission tomography. ^1^ CRE-1 performed 4–6 weeks after completion of nCRT; CRE-2 performed 10–12 weeks after completion of nCRT. ^2^ EUS FNA lymph node positive (n = 8); nonrepresentative material (n = 6); uncertain cytology (n = 1); EUS not performed (n = 1). ^3^ EUS nonrepresentative material (n = 3). ^4^ EGD with bite-on-bite biopsies uncertain (n = 1).		

**Table:** **Table 4** Detection of locoregional regrowth in patients undergoing active surveillance at each clinical response evaluation (CRE).

CRE number (time after completion of nCRT, months ^1^	Patients with recurrent disease (n = 132) and the diagnostic methods for detection of locoregional regrowth ^2^
3 (6)	Locoregional regrowth (n = 49) ^3^ Local: EGD with bite-on-bite biopsies (n = 44) PET positive (n = 38) ^4^ PET negative (n = 5) Detected by EGD only (n = 1) Local: PET (n = 1) PET primary tumor positive, EUS negative, EGD no biopsies Regional: EUS (n = 4) LNs detected by EUS + FNA, PET LN positive ^5^
	Metastases (n = 21) ^6^
4 (9)	Locoregional regrowth (n = 20) Local: EGD with bite-on-bite biopsies (n = 18) PET positive (n = 13) ^7^ PET negative (n = 5) Local: EUS/PET (n = 2) Primary tumor site detected by EUS + FNB, PET positive (n = 1) Uncertain EGD pathology, EUS suspicious, PET positive (n = 1)
5 (12)	Locoregional regrowth (n = 13) Local: EGD with bite-on-bite biopsies (n = 10) PET positive (n = 5) PET negative (n = 3) ^8^ Suspicious EGD, pathology negative, PET positive (n = 1) No pass, PET positive (n = 1) Regional: EUS/PET (n = 3) LNs detected by EUS + FNA, PET LN positive (n = 1) LNs detected by EUS + FNA, PET LN negative (n = 1) LNs detected by PET, EUS not performed (n = 1)
	Metastases (n = 4) ^9^
6 (16)	Locoregional regrowth (n = 4) Local: EGD with bite-on-bite biopsies (n = 3) PET positive (n = 3) Regional: LNs detected by PET, not reachable for FNA (n = 1)
	Metastases (n = 2)
7 (20)	Metastases (n = 1)
8 (24)	Locoregional regrowth (n = 4) Local: EGD with bite-on-bite biopsies (n = 4) PET negative (n = 3) ^10^ PET positive (n = 1)
9 (30)	Locoregional regrowth (n = 2) Local: EGD with bite-on-bite biopsies (n = 2) PET positive (n = 2)
	Metastases (n = 3)
10 (36)	Locoregional regrowth (n = 8) Local: EGD with bite-on-bite biopsies (n = 8) PET positive (n = 4) ^11^ PET negative (n = 4)
	Metastases (n = 1)
**Summary of detection methods for the 100 cases of locoregional regrowth**	
Local EGD with bite-on-bite biopsies (n = 87) EUS + FNB, PET primary tumor site positive (n = 1) PET with EGD and EUS having no added value (n = 1) Combination of EGD (without pathologic confirmation) and PET (n = 3) Regional LNs detected by EUS + FNA, PET LN positive (n = 5) LNs detected by EUS + FNA, PET LN negative (n = 1) LNs detected by PET with EUS having no added value (n = 2)	
EGD, esophagogastroduodenoscopy; bob: bite-on-bite biopsy; EUS, endoscopic ultrasound; FNA, fine-needle aspiration; LN, lymph node; PET, positron emission tomography. ^1^ No patients with recurrent disease were detected at CRE-11 (48 months) or CRE-12 (60 months). ^2^ In addition, four patients who declined active surveillance developed metastases, 13 patients died without disease recurrence, and 49 patients had a sustained complete clinical response (not included in this table). ^3^ Perioperative metastasis found (n = 1). ^4^ EUS not performed (n = 1); EUS FNA LN or primary tumor positive (n = 1); EUS uncertain (n = 3), including nonrepresentative (n = 1); EUS no pass (n = 3). ^5^ LNs detected with EUS and positive PET but found to have perioperative metastases (n = 1); EGD with uncertain biopsies (n = 1). ^6^ Locoregional regrowth detected by EGD with bite-on-bite biopsies but metastases detected within 2 weeks (n = 1). ^7^ EUS suspicious but no FNA (n = 1); EUS no pass (n = 1); EUS not performed (n = 2). ^8^ EUS uncertain (n = 1). ^9^ Oligometastasis found, continued active surveillance after treatment and had locoregional relapse detected by EGD with bite-on-bite biopsies at CRE-7 (n = 1). ^10^ EUS showed suspicious LNs that could not be reached (n = 1). ^11^ EUS not performed (n = 1).	

At CRE-2, EGD with bite-on-bite biopsies detected local regrowth in 101/457 patients (22%, 95%CI 18%–26%), representing 101/114 patients with local regrowth (89%, 95%CI 81%–94%). During active surveillance, the majority of patients with regrowth (87/100; 87%, 95%CI 79%–93%) were also identified by EGD with bite-on-bite biopsies, corresponding to a diagnostic yield of 18% (70/388; 95%CI 14%–22%) in the first year beyond CRE-2. In the second year, the yield was 2.9% (7/243; 95%CI 1.2%–5.8%), in the third year 7.6% (10/132; 95%CI 3.7%–13.5%) and no regrowth was detected in years 4 and 5 with the current follow-up.

At CRE-2, EUS with FNA identified five patients with lymph node metastases and FDG uptake on PET-CT (5/114; 4.4%, 95%CI 1.4%–9.9%) and seven patients without FDG uptake in the lymph node metastases (7/114; 6.1%, 95%CI 2.5%–12.2%). During active surveillance, one patient with local regrowth was detected by EUS with FNB, with PET-CT showing increased FDG uptake at the primary tumor site (1/100; 1.0%, 95%CI 0.0%–5.5%) ( **Appendix 1s** ). During active surveillance, EUS with FNA detected six patients with lymph node metastases (6/100; 6.0%, 95%CI 2.2%–12.6%): five lymph nodes were FDG positive on PET-CT, and one lymph node located in the celiac trunk (CRE-5; 12 months after nCRT) was FDG negative.

At CRE-2, one patient with a lymph node metastasis was detected by FDG uptake on PET-CT, which was confirmed by endoscopic bronchial ultrasound (EBUS) with FNA (1/114; 0.9%, 95%CI 0.0%–4.8%). During active surveillance, PET-CT alone detected 3/100 patients with a high suspicion of residual tumor without pathologic confirmation (3.0%, 95%CI 0.6%–8.5%): one patient with local regrowth and two patients with lymph node metastases. In all three patients, neither EGD or EUS provided additional diagnostic value, with all three undergoing resection, which confirmed locoregional regrowth.

Lastly, during active surveillance, three patients with local regrowth (confirmed in the resection specimen) were identified through a combination of EGD (without pathologic confirmation) and PET-CT.

### Hypothetical benefit of EUS for suspicious lymph nodes on PET-CT

#### CRE-2

In a hypothetical cohort of 1000 patients at CRE-2, 220 patients would be identified with local regrowth by EGD with bite-on-bite biopsies (22%; 101/466) and 80 patients with distant metastases by PET-CT (8.2%; 38/466) ( Fig. 1 ), thereby excluding 300 patients from the need to undergo EUS. Of the remaining 700 patients, only 56 patients would undergo EUS based on PET-CT suspicion of regional lymph node involvement (8.0%; 26/327) ( Table 2 ), thereby allowing 644 patients to omit the procedure (92%; 644/700). This selective strategy would miss 21 patients with regional lymph node metastases (2.1%; 7/327) ( Table 2 ). Based on this selective strategy, the NNS with EUS to detect one additional patient with regional lymph node metastasis missed by PET-CT is 5 (26/5) compared with 65 (327/5) when considering the full cohort.

#### Active surveillance

During active surveillance (beyond CRE-2) in a hypothetical cohort of 1000 patients with CCR after nCRT, performing EUS based on PET-CT indication would result in five missed patients with regional lymph node metastases (0.5%, 95%CI 0.0%–2.8%; 1/198) ( Table 4 ). This approach would however allow 97.8% of EUS procedures to be omitted, with only 2.2% of EUS procedures being necessary based on PET-CT suspicion ( Table 2 ). In the first year of active surveillance (beyond CRE-2), the percentage of EUS procedures performed based on PET-CT findings would be 3.7%. This percentage would be expected to decrease further in the second year to 1.4%, followed by a reduction to 0.8% in the third year, and ultimately to 0% after the fourth year ( **Table 3s** ). This approach would save 95.7% (704/736) of all EUS procedures in the first 3 years of active surveillance (complete 3-year follow-up in the SANO cohort).

## Discussion

During active surveillance, 25% of patients (49/198) had a sustained clinical complete response (CCR), 7% (13/198) died without disease recurrence, and 18% (36/198) developed distant metastases, including 11% (21/198) within 6 months after nCRT (minimum follow-up of 3 years). Esophagectomy was arguably rightfully withheld in 43% of patients. Locoregional regrowth occurred in 51% (100/198). Although these patients could be safely operated on, delayed detection might increase the risk of metastases and adversely affect outcomes. Early and accurate detection of locoregional regrowth is therefore important to mitigate these potential downsides. We found that, for this purpose, the role of EUS appears to be limited: 87% of patients with locoregional regrowth were primarily detected by EGD with bite-on-bite biopsies, 3% by PET-CT, and 1% by EUS ( Table 4 ).

The 1% detected by EUS corresponds to regional lymph nodes missed in 0.1% of all PET-CT scans, affecting 0.5% of patients (1/198) during active surveillance. Most patients with residual disease were detected through EGD with bite-on-bite biopsies during the first 3 months after nCRT; however, at CRE-2, regrowth in regional lymph nodes would have been missed by PET-CT in 2.1%, leading to undetected regional lymph node metastases in seven patients. These findings suggest that selective use of EUS with FNA based on PET-CT findings during active surveillance (beyond CRE-2) may be a reasonable approach in patients with esophageal cancer and CCR after nCRT.

The likelihood of detecting locoregional regrowth declines as time from nCRT increases, particularly for regional lymph nodes. Therefore, standard EUS with FNA could be considered for performance only at CRE-2, when the a priori chance of detecting residual tumor is higher. From 6 months post-nCRT (CRE-3) onward, EUS with FNA could be reserved for patients with lymph nodes that are positive on PET-CT to reduce the number of nonbeneficial procedures during active surveillance; however, this implies that, in 0.5% of patients (1/198), a tumor positive lymph node would be missed. This undetected lymph node could progress and lead to irresectable disease or theoretically give rise to other metastases and as such increase the risk for cancer-related mortality.

Acceptable accuracy rates for diagnostic tests and NNS vary by context and should be interpreted with caution. By analogy, missing nodal disease in esophageal cancer may affect survival, supporting a cautious approach to omitting EUS. But patients undergoing active surveillance would be spared a total of 704 EUS procedures in the first 3 years after nCRT ( **Table 4s** ). In our cohort, this approach could have saved €743368 (calculated at a unit price of €1056 per EUS, based on Benchmark costs for 2023) 10 . Additionally, it would reduce the workload in the endoscopy departments and alleviate the burden on patients by minimizing invasive procedures. Although EGD and EUS are performed in the same session, EUS adds procedure time, may require deeper sedation, and involves a larger scope. Therefore, if EUS is performed when indicated, scheduling PET-CT and EGD on the same day could further minimize patient burden.

Our results align with previous research. In the preSANO trial, it was noted that PET-CT failed to detect 15% of cases of tumor regression grade 3–4 locoregional regrowth, while the combination of bite-on-bite biopsies and EUS-FNA improved diagnostic sensitivity 3 . In the preSINO trial, which included only patients with squamous cell carcinoma, EUS-FNA detected residual tumor in an additional 21% of patients with negative biopsies in the subgroup with tumor regression grade 1–2 and ypN+ disease (6/29) 11 . Notably, lymph node metastases are more prevalent in squamous cell carcinoma compared with adenocarcinoma, which may explain the higher diagnostic yield of EUS-FNA in that setting 12 .

Cerfolio et al. 13 , in a prospective study, compared EUS-FNA, PET-CT, and CT performance in post-nCRT restaging of patients with esophageal cancer. PET-CT was most accurate for nodal involvement (93%), compared with EUS-FNA (78%) and CT (78%). Also, PET-CT correctly predicted complete response in 89% of patients compared with 67% with EUS-FNA.

Similar information is available regarding PET-CT accuracy when applied to tumors in different anatomical regions. In rectal cancer, the study by Perez et al. 14 , showed that post-CRT PET was 93% sensitive and nonspecific (53%) for complete response but, when added to clinical assessment, PET raised overall diagnostic accuracy from 91% to 96%. This is highly applicable to our setting, where PET alone may fail to detect isolated nodal regrowth.

Diagnostic CT may not provide additional value over PET-CT in the post-nCRT evaluation, as studies show that PET-CT alone is sufficiently accurate for detecting lymph node metastases, even though its CT component is typically low dose and of lower quality than a standalone diagnostic CT scan 15 16 . Moreover, ^18^ F-FDG PET/MRI showed no improvement in tumor detection compared with ^18^ F-FDG PET-CT in tumor detection following nCRT 17 . Radiomics is emerging as a promising noninvasive approach for diagnosis, with deep learning models showing promising accuracy in predicting lymph node metastasis 18 . Our ^18^ F-FDG PET-based model for detecting residual tumor post-nCRT did not however demonstrate clinical utility 19 .

This study has several limitations. First, prior PET-CT and EUS findings may have influenced physician discretion, as previously negative or nonsuspicious results could have reduced the likelihood of performing a subsequent FNA. This may have led to missed patients with lymph node metastases or local regrowth, underestimating the diagnostic value of EUS-FNA. Positive lymph nodes were defined based on radiologist interpretation rather than a standardized uptake value (SUV) cutoff, which may have introduced interobserver variability and limit reproducibility across centers. Additionally, the a priori chance of detecting a positive lymph node decreases over time after nCRT, which may lead to an underestimation of the diagnostic sensitivity of EUS-FNA, as the lower prevalence of residual disease reduces the likelihood of detecting true positives. In addition, the relatively low rate of lymph node metastases in active surveillance prevented us from performing a statistically meaningful subgroup analysis to investigate the differential PET and EUS performance according to nodal stations, which is important because test accuracy may vary by location.

Another limitation of this study is the absence of detailed data on the intensity of FDG uptake in the primary tumor at baseline. Baseline FDG uptake is important, as tumors with lower uptake may be harder to detect by PET-CT during follow-up. Inclusion in the SANO trial required FDG-avid tumors, including those with moderate FDG avidity. As such, the findings may also be applicable to patients with moderately FDG-avid tumors.

Furthermore, we used EUS-FNA as the reference standard; however, this is not without limitations. Because the exact locations of lymph nodes in the resection specimen were not reported and not all patients were operated on, we were unable to calculate a false-negative rate for EUS-FNA. Lastly, the generalizability of our findings may be limited by the high volume, specialized centers in which all procedures were performed by experienced operators. Tumor histology and location can also influence diagnostic performance, as differences in lymphatic spread and esophageal anatomy may affect EUS-FNA detection of residual nodal disease and PET-CT accuracy. These factors highlight the potential value of tailoring surveillance strategies to maximize diagnostic yield, while minimizing unnecessary procedures.

Despite these limitations, the large sample size, multicenter design, and real-world applicability of our findings are of particular interest with regard to the optimal use of PET-CT and EUS in post-nCRT surveillance of esophageal cancer. Future studies should perform a complete cost-effectiveness analysis of different surveillance strategies. EGD with bite-on-bite biopsies remains the cornerstone of surveillance for detecting locoregional regrowth, but its diagnostic yield should be interpreted with caution, as only confirmed true positives are known and true negatives cannot be reliably determined without surgery. With most patients followed for 4 years, our data suggest that the risk of locoregional regrowth substantially decreases after 3 years post-nCRT; however, as 7.6% of patients developed regrowth in the third year of follow-up, the current follow-up strategy seems justified for the first 3 years, with the potential to reduce intensity in years 4 and 5, pending further confirmation.

Our findings confirm that PET-CT is insufficient to exclude nodal involvement and that EUS-FNA, when selectively applied, does have valuable diagnostic information to offer. Based on these results, a multimodal adaptive approach appears most reasonable: EGD should be used for primary tumor site surveillance, PET-CT for interval metastases, while EUS could be selectively applied in the setting of questionable uptake on PET-CT during active surveillance (after 3 months post-nCRT), where the a priori chance of detecting locoregional regrowth is lower. This approach may optimize diagnostic yield, reduce inappropriate invasive testing, and adhere to a patient-focused cost-effective paradigm of care.

## Supplementary Material

Supplementary Material Zusatzmaterial
