# Supplementary material for: The value of routine endoscopic ultrasound in patients with esophageal cancer undergoing active surveillance after neoadjuvant chemoradiotherapy
Source: Endoscopy. 2026 Feb 12;58(6):577–87. doi: 10.1055/a-2776-5896 (PMC13305576; doi:10.1055/a-2776-5896)
Supplement: Supplementary file 1 — Supplementary Material [file 10-1055-a-2776-5896_28005943.pdf]

Supplementary material

The value of routine endoscopic ultrasound in patients with esophageal cancer undergoing active surveillance after neoadjuvant chemoradiotherapy

Sanjiv S. G. Gangaram Panday, Matteo Pittacolo, Sjoerd M. Lagarde, Bianca Mostert, Judith Honing, J. Jan B. van Lanschot, Tanya M. Bisseling, Erik J. Schoon, Jolanda M. van Dieren, Rutger Quispel, Liekele E. Oostenbrug, Andries van der Linden, Sietske Corporaal, Lieke Hol, Eva Kouw, Jurjen J. Boonstra, Wouter L. Hazen, Erik Vegt, Manon C. W. Spaander, Bas P. L. Wijnhoven; on behalf of the SANO study group

Contents **Page**

Table 1s: Members of the SANO study group **2**

Table 2s: Number of patients with PET-CT and EUS at each clinical response evaluation **5**

Appendix 1s: Fine needle biopsy (FNB) during clinical response evaluations **5**

Table 3s: PET-CT lymph node uptake vs EUS (with FNA) outcome for lymph nodes at CRE-2 and during active surveillance, prior to excluding those with regrowth already detected by endoscopy or with distant metastases **6**

Table 4s: PET-CT suspicious lymph nodes per year after nCRT in all procedures and with exclusion of EGD positives and metastasis detected by PET-CT **7**

Endoscopy | DOI 10.1055/a-2776-5896 | © The Author(s).

Table 1s: Additional members of the SANO study group, not listed among the authors.

Name	Department	Centre
Arjun D. Koch	Gastroenterology	Erasmus MC – University medical center
Suzan Nikkessen	Gastroenterology	Erasmus MC – University medical center
Ate van der Gaast	Medical oncology	Erasmus MC – University medical center
Roelf Valkema	Nuclear medicine	Erasmus MC – University medical center
Michail Doukas	Pathology	Erasmus MC – University medical center
Lindsey Oudijk	Pathology	Erasmus MC – University medical center
Hester F. Lingsma	Public health	Erasmus MC – University medical center
David van Klaveren	Public health	Erasmus MC – University medical center
Roy S. Dwarkasing	Radiology	Erasmus MC – University medical center
Berend van der Wilk	Surgery	Erasmus MC – University medical center
Ben Eyck	Surgery	Erasmus MC – University medical center
Bo Jan Noordman	Surgery	Erasmus MC – University medical center
Maartje Valkema	Surgery	Erasmus MC – University medical center
Geert J. Bulte	Gastroenterology	Radboud University Medical Centre
Peter D. Siersema	Gastroenterology	Radboud University Medical Centre
Harm Westdorp	Medical oncology	Radboud University Medical Centre
Erik H. Aarntzen	Nuclear medicine	Radboud University Medical Centre
Chella S. van der Post	Pathology	Radboud University Medical Centre
Maartje C. van Rijk	Radiology	Radboud University Medical Centre
Pètra M. Braam	Radiotherapy	Radboud University Medical Centre
Heidi Rütten	Radiotherapy	Radboud University Medical Centre
Marcel Verheij	Radiotherapy	Radboud University Medical Centre
Camiel Rosman	Surgery	Radboud University Medical Centre
Bastiaan Klarenbeek	Surgery	Radboud University Medical Centre
Serge J. Zweers	Gastroenterology	Maasstad Hospital
Lisanne Holster	Gastroenterology	Maasstad Hospital
Ewout F.W. Courrech Staal	Radiology	Maasstad Hospital
Karen E. Hamoen	Pathology	Maasstad Hospital
Trudy Rapmund	Surgery	Maasstad Hospital
Erwin van der Harst	Surgery	Maasstad Hospital
Peter-Paul Coene	Surgery	Maasstad Hospital
Peter Oosterwijk	Gastroenterology	Ziekenhuisgroep Twente Hospital
Polat Dura	Gastroenterology	Ziekenhuisgroep Twente Hospital
Ronald Hoekstra	Medical oncology	Ziekenhuisgroep Twente Hospital
Ali Agool	Nuclear medicine	Ziekenhuisgroep Twente Hospital
Joop van Baarlen	Pathology	Ziekenhuisgroep Twente Hospital
Ellen M. Hendriksen	Radiotherapy	Ziekenhuisgroep Twente Hospital
Henk Jan Mantel	Surgery	Ziekenhuisgroep Twente Hospital
Marc van Det	Surgery	Ziekenhuisgroep Twente Hospital
Ewout Kouwenhoven	Surgery	Ziekenhuisgroep Twente Hospital
Sana A. Mulder	Gastroenterology	Reinier de Graaf Gasthuis
Arjan J. Verschoor	Medical oncology	Reinier de Graaf Gasthuis

Endoscopy | DOI 10.1055/a-2776-5896 | © The Author(s).

Marc R.J. ten Broek	Nuclear medicine	Reinier de Graaf Gasthuis
René J. Dallinga	Radiology	Reinier de Graaf Gasthuis
Karen J. Neelis	Radiotherapy	Reinier de Graaf Gasthuis
Erlinde de Graaf	Surgery	Reinier de Graaf Gasthuis
Stijn van Esser	Surgery	Reinier de Graaf Gasthuis
Jan Willem Dekker	Surgery	Reinier de Graaf Gasthuis
Thomas R. de Wijkerslooth	Gastroenterology	The Netherlands Cancer Institute
Marieke A. Vollebergh	Medical oncology	The Netherlands Cancer Institute
Emilia C. Owers	Nuclear medicine	The Netherlands Cancer Institute
Annemarieke Bartels-Rutten	Radiology	The Netherlands Cancer Institute
Liudmila L. Kodach	Pathology	The Netherlands Cancer Institute
Francine E.M. Voncken	Radiotherapy	The Netherlands Cancer Institute
Yvonne Hilhorst	Surgery and gastroenterology	The Netherlands Cancer Institute
Marjolein Warmerdam	Surgery and gastroenterology	The Netherlands Cancer Institute
Johanna van Sandick	Surgery	The Netherlands Cancer Institute
Edward Fiets	Medical oncology	Medical Centre Leeuwarden
Marco B. Polée	Medical oncology	Medical Centre Leeuwarden
Anne Marij G. van Burg	Nuclear medicine	Medical Centre Leeuwarden
Judith Nieken	Pathology	Medical Centre Leeuwarden
Rinze Wolf	Radiology	Medical Centre Leeuwarden
Vera Oppedijk	Radiotherapy	Medical Centre Leeuwarden
Marloes Emous	Surgery	Medical Centre Leeuwarden
Daniel A. Hess	Surgery	Medical Centre Leeuwarden
Jean Pierre Pierie	Surgery	Medical Centre Leeuwarden
Willemien Erkelens	Gastroenterology	Gelre Hospital
S. Cathrien S. Tromp – van Driel	Medical oncology	Gelre Hospital
Marc D. Zuijdwijk	Nuclear medicine	Gelre Hospital
H. Doornewaard	Pathology	Gelre Hospital
Karin Muller	Radiotherapy	Gelre Hospital, Radiotherapiegroep Deventer
Peter van Duijvendijk	Surgery	Gelre Hospital
Eelco B. Wassenaar	Surgery	Gelre Hospital
Edwin van der Zaag	Surgery	Gelre Hospital
Geert-Jan Creemers	Medical oncology	Catharina Hospital
Mark J. Roef	Nuclear medicine	Catharina Hospital
Ineke van Lijnschoten	Pathology	Catharina Hospital
Joost Nederend	Radiology	Catharina Hospital
Maurice J.C. van der Sangen	Radiotherapy	Catharina Hospital
Tom C.G. Budiharto	Radiotherapy	Catharina Hospital
Fanny F.B.M. Heesakkers	Surgery	Catharina Hospital
Misha Luyer	Surgery	Catharina Hospital
Grard Niewenhuijzen	Surgery	Catharina Hospital
Fabienne A.R.M. Warmerdam	Medical oncology	Zuyderland Medical Centre
Wendy Schreurs	Nuclear medicine	Zuyderland Medical Centre

Endoscopy | DOI 10.1055/a-2776-5896 | © The Author(s).

Robert Riedl	Pathology	Zuyderland Medical Centre
Roy F.A. Vliegen	Radiology	Zuyderland Medical Centre
Jeroen Buijsen	Radiotherapy	Zuyderland Medical Centre
Ilse Stohr	Surgery	Zuyderland Medical Centre
Eric H.J. Belgers	Surgery	Zuyderland Medical Centre
Meindert Sosef	Surgery	Zuyderland Medical Centre
Marije Slingerland	Medical oncology	Leiden University Medical Centre
Richard Raghoo	Nuclear medicine	Leiden University Medical Centre
A. Stijn L.P. Crobach	Pathology	Leiden University Medical Centre
Aart J. van der Molen	Radiology	Leiden University Medical Centre
Susan J.C.L.M. Quix	Surgery	Leiden University Medical Centre
Wobbe O. de Steur	Surgery	Leiden University Medical Centre
Henk Hartgrink	Surgery	Leiden University Medical Centre
Laurens V. Beerepoot	Medical oncology	Elisabeth Tweesteden Hospital
David E. Ploeg	Pathology	Elisabeth Tweesteden Hospital
Tom Rozema	Radiotherapy	Elisabeth Tweesteden Hospital
Ilse A.C. Vermeltfoort	Nuclear medicine	Elisabeth Tweesteden Hospital
Walther Jansen	Surgery	Elisabeth Tweesteden Hospital
Joos Heisterkamp	Surgery	Elisabeth Tweesteden Hospital

Endoscopy | DOI 10.1055/a-2776-5896 | © The Author(s).

Table 2s: Number of patients with PET-CT and EUS combination and EGD with biopsies at each clinical response evaluation.

CRE (months after nCRT)	EUS + PET-CT	EUS + PET-CT[2]	EGD with biopsies
1 (1.5)	-	-	**758**
2 (3)	450	327	457
3 (6)	174	119	183
4 (9)	110	95	112
5 (12)	90	82	93
6 (16)	81	75	80
7 (20)	83	82	92
8 (24)	70	64	71
9 (30)	63	60	65
10 (36)	65	58	67
11 (48)	39	37	41
12 (60)	7	7	7
Total	1232	1006	2026

CRE: clinical response evaluation; nCRT: neoadjuvant chemoradiotherapy; EUS: endoscopic ultrasound; EGD: esophagogastroduodenoscopy.
[2]after exclusion of esophagogastroduodenoscopy with positive biopsies and distant metastasis

Appendix 1s: Fine needle biopsy (FNB) during clinical response evaluations.

In our cohort, EUS-FNB was performed and documented three times before resection: once during CRE-2 (for a submucosal lesion, no tumor detected) and twice during active surveillance. During active surveillance, one patient had residual tumor detected in the esophageal wall by FNB, with concurrent increased FDG uptake on the PET scan and negative biopsies. In the other patient, a suspicious lymph node was evaluated with both FNA and FNB, but no residual tumor was detected and there was no FDG uptake on the PET scan. Additionally one EUS-FNB was performed after local regrowth and oesophagectomy to confirm a lymph node metastasis at the celiac axis.

Endoscopy | DOI 10.1055/a-2776-5896 | © The Author(s).

Table 3s: PET-CT lymph node uptake vs EUS (with FNA) outcome for lymph nodes at CRE-2 and during active surveillance, prior to excluding those with regrowth already detected by endoscopy or with distant metastases.

**All PET + EUS**	EUS + FNA positive	EUS negative	Total
PET LN positive*	23 (1.9)	55 (4.5)	78
PET LN negative	**12 (1.0)**	1142 (92.7)	1154
Total	35	1197	1232
**CRE-2**	EUS + FNA positive	EUS negative	Total
PET LN positive	13 (2.9)	32 (7.1)	45
PET LN negative	**9 (2.0)**	396 (88.0)	405
Total	22	428	450
**Active surveillance**	EUS + FNA positive	EUS negative	Total
PET LN positive	10 (1.3)	23 (2.9)	33
PET LN negative	**3 (0.4)**	746 (95.4)	749
Total	13	769	782

EUS: endoscopic ultrasound; LN: lymph node;
*PET LN positive indicates suspicion
Assumed negative as EUS did not add in detection (PET suspect LN no/yes):
- 24 no FNA performed with suspect lymph nodes (12 no, 10 yes)
- 42 non-representative material (38 no, 4 yes)
- 4 uncertain cytology (4 no)

Endoscopy | DOI 10.1055/a-2776-5896 | © The Author(s).

Table 4s: PET-CT suspicious lymph nodes per year after nCRT in all procedures and with exclusion of EGD positives and metastasis detected by PET-CT.

CRE (months after nCRT)	PET LN	n (%)	n (%)[2]
3-5 (6-12)	No<br>Yes	348 (93)<br>26 (7)	285 (96.3)<br>11 (3.7)
6-8 (16-24)	No<br>Yes	230 (98)<br>4 (1.7)	218 (98.6)<br>3 (1.4)
9-10 (30-36)	No<br>Yes	126 (98.4)<br>2 (1.6)	117 (99.2)<br>1 (0.8)
11-12 (48-60)	No<br>Yes	45 (97.8)<br>1 (2.2)	44 (100)<br>0
Total		782	679

CRE: clinical response evaluation; nCRT: neoadjuvant chemoradiotherapy; LN: Lymph node.
[2]after exclusion of esophagogastroduodenoscopy with positive biopsies and distant metastasis

Endoscopy | DOI 10.1055/a-2776-5896 | © The Author(s).
